# Vision screening using a smartphone platform

**DOI:** 10.1590/1984-0462/2022/40/2020021IN

**Published:** 2022-05-06

**Authors:** Iara Debert, Douglas Rodrigues da Costa, Mariza Polati, Janaina Guerra Falabretti, Remo Susanna

**Affiliations:** a Universidade de São Paulo, São Paulo, SP, Brazil.

**Keywords:** Vision screening, Amblyopia, Refractive errors, Seleção visual, Ambliopia, Erros de refração.

## Abstract

**Objective:**

The main aim of this study was to evaluate the performance of a platform designed for pediatricians to screen amblyopia using a smartphone.

**Methods:**

The medical records of consecutive children who received visual screening using a smartphone platform were retrospectively reviewed. The smartphone was used with a flash concentrator case and a software for capturing images of both eyes simultaneously by a photorefraction mechanism. The platform performance was compared to the comprehensive ophthalmological examination, which is considered the gold standard for detecting amblyopia. Sensitivity, specificity, positive predictive value, and negative predictive value of the software in detecting amblyopia risk factors were calculated.

**Results:**

A total of 157 children were included, with a mean age of 6.0±.5 years (range 5–7). In 94% of the cases, the software was able to analyze the images and release a result, determining whether or not the child presented with amblyopia risk factors. Compared to the ophthalmological examination, the smartphone platform sensitivity in detecting amblyopia risk factors was 84%, the specificity was 74%, the positive predictive value was 86%, and the negative predictive value was 70%.

**Conclusions:**

The sensitivity and specificity of the smartphone photoscreening platform to detect amblyopia risk factors were within the range of traditional instrument-based vision screening technology. A smartphone photorefraction platform appears to be a promising cost-effective alternative to assist pediatricians and minimize obstacles to vision screening and amblyopia detection. Future studies are needed to gather additional comparative data.

## RESUMO

**Objetivo:**

Avaliar a performance de uma plataforma desenvolvida para pediatras para triagem de ambliopia utilizando um smartphone.

**Métodos:**

Os prontuários consecutivos de crianças submetidas a triagem visual usando uma plataforma no smartphone foram analisados retrospectivamente. Uma capa concentradora de flash foi utilizada no smartphone com um software para capturar imagens simultâneas dos dois olhos por um mecanismo de fotorrefração. A performance da plataforma foi comparada ao exame oftalmológico completo, considerado o padrão ouro para detecção de ambliopia. Foram calculados sensibilidade, especificidade, valor preditivo positivo e valor preditivo negativo do software em detectar fatores de risco para ambliopia.

**Resultados:**

Foram incluídas 157 crianças com idade média de 6.0±0.5 anos (variação de 5 a 7 anos). Em 94% dos casos, o software foi capaz de analisar as imagens e fornecer um resultado, determinando se a criança apresentava ou não fatores de risco para ambliopia. Comparados ao exame oftalmológico, a sensibilidade da plataforma no smartphone em detectar fatores de risco para ambliopia foi de 84%, a especificidade foi de 74%, o valor preditivo positivo foi de 86% e o valor preditivo negativo foi de 70%.

**Conclusões:**

A plataforma de triagem por fotorrefração usando o smartphone apresentou sensibilidade e especificidade para detectar fatores de risco para ambliopia semelhantes às encontradas em instrumentos tradicionais de triagem por fotorrefração. Uma plataforma no smartphone é uma alternativa custo-efetiva promissora para auxiliar pediatras a minimizar os obstáculos para triagem visual e detecção da ambliopia. Estudos futuros são necessários a fim de reunir dados comparativos adicionais.

## INTRODUCTION

The main goal of vision screening in childhood is to detect amblyopia, the leading cause of monocular vision impairment in children and young adults.^1,2^ Amblyopia is defined as reduced best-corrected visual acuity caused by abnormal visual development. Adequate visual maturation requires the brain to obtain images from both eyes simultaneously and with similar clarity. Anything that interferes with the visual pathways development during the critical period of visual maturation may cause amblyopia, as a result of inadequate stimulation of the visual cortex.^1,3^ The main causes are refractive errors and strabismus. Although visual loss from amblyopia is preventable and can be successfully treated in childhood, permanent and irreversible visual loss results if amblyopia is not diagnosed and treated early.^4^

Visual acuity testing is the traditional and most widely used method for amblyopia detection in verbal children. It can be performed as vision screening by schoolteachers or pediatricians. It is a subjective measurement and requires participation by the child to identify optotypes such as shapes, symbols, or letters using each eye separately.^5,6,7^ For preverbal children, visual acuity and amblyopia can be accessed by preferential looking tests (e.g., Teller Acuity Card Test and Lea Gratings), fixation preference test in patients with eye misalignment, and electrophysiological testing using sweep visual evoked potential. For screening purposes in preverbal children or for those who cannot cooperate with monocular visual acuity measurement, detection of amblyopia risk factors can be done by photorefraction, an instrument-based screening that identifies the main risk factors for amblyopia: hyperopia (farsightedness), myopia (nearsightedness), astigmatism (difference in refractive errors between the ocular meridians), strabismus (eye misalignment), and anisometropia (difference in refractive errors between the eyes). Photoscreeners are flash cameras that work by shining light at both eyes simultaneously to produce a red reflex in the pupils. The instrument has a software that analyzes the light crescents related to defocus in the generated images to determine whether the child passes or should be referred for ophthalmological evaluation.^8,9^ Because early detection of amblyopia generally produces better treatment outcomes, vision screening has been recommended using photorefraction in young children.^10,11^

More recently, photorefraction has also been performed using a smartphone platform. The GoCheck Kids software (Gobiquity Mobile Health, Scottsdale, AZ, USA) is a smartphone photoscreener designed for pediatricians to detect amblyopia risk factors and has been registered with the U.S. Food and Drug Administration (FDA). The first version of the software was able to detect hyperopia, myopia, and anisometropia, but not astigmatism, because the refractive error was measured in one meridian only.^12,13,14,15,16^ A new software version was recently designed to detect astigmatism and improve accuracy. The purpose of this study was to evaluate the performance of the GoCheck Kids screener in its newest version in detecting amblyopia risk factors.

## METHOD

The medical records of consecutive children who received photoscreening using the GoCheck Kids platform were retrospectively reviewed. All children underwent a comprehensive ophthalmological evaluation at the Hospital das Clinicas of the University of Sao Paulo during the Visão do Futuro (“Vision of the Future”) social program. In this program, children are previously screened at São Paulo public schools, where local teachers perform monocular visual acuity measurements using an optotype eye chart (Snellen chart). All the children who fail the local screening in one or both eyes are referred to specialized ophthalmological evaluation. The study was approved by the Institutional Review Board of the Hospital das Clinicas of the University of Sao Paulo (approval number 29047520.9.0000.0068). Both the photoscreening and the ophthalmological examination were performed in the same day and included visual acuity test using Snellen chart, motility evaluation with cover test, anterior segment evaluation with slit lamp, cycloplegic refraction with cyclopentolate 1% drops, and fundus examination. Children who did not have a comprehensive eye examination were excluded.

**Figure 1.**
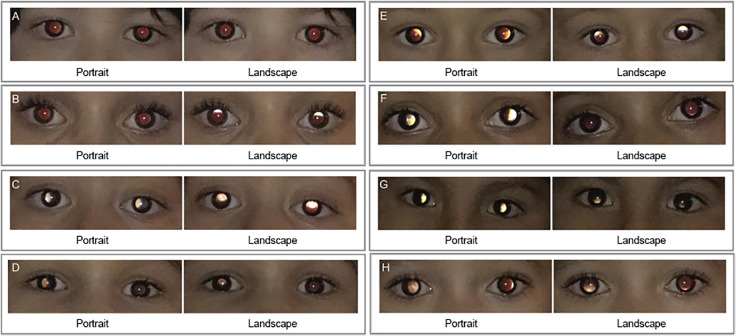
Results displaying amblyopic risk factors in eight representative patients. No risk factors identified (A). Myopic astigmatism (B). Myopia (C). Myopic anisometropia (D). Astigmatism (E). Hyperopic astigmatism (F). Hyperopia (G). Hyperopic anisometropia (H).

Photoscreening was performed prior to the instillation of cycloplegic drops. The GoCheck Kids platform was used on an Apple iPhone 7 (Apple Inc., Cupertino, CA, USA) with a flash concentrator case for photorefraction and a software for capturing and processing the images. In a dimly lit room, two photographs were taken, the first with the phone in portrait mode (vertical meridian) and the second in landscape mode (horizontal meridian). To attract the child’s attention at a distance of approximately 0.7m, the phone makes animal noises to take a picture of both eyes simultaneously in a few seconds. References are displayed on the screen to optimize alignment and to obtain the appropriate distance. The examiner reviewed the images to ensure the child was looking at the camera before accepting the photo. If the eyes were too far or too close, an error message was shown, and the picture was retaken. Images were then processed using the application algorithm, which identifies and creates estimates of eye metrics to calculate the photorefraction value. Anisometropia (difference in refractive errors between the eyes) was displayed if there was a significant difference in the refractive errors between the eyes. Strabismus was suspected based on the position of the corneal light reflex relative to the pupil. The values were compared to the application’s referral criteria for amblyopic risk factors, and an immediate result was displayed automatically (Figure 1). The results were displayed as “risk factors identified,” “no risk factors identified at this time,” or “not gradable,” referring to an inadequate photograph. The main reasons for not gradable images are the photo was taken too close or too far from the eyes, the child did not look directly at the camera, the pupils were too small due to a bright area, there were spurious corneal reflections, or the child was moving when the picture was taken. Images were automatically uploaded to the application database, where they were reviewed remotely by trained ophthalmic imaging specialists. When the review generated a change in the result, the examiner received a feedback to update the recommendation. The updated results were included in the data analysis. Both the examiner performing the photoscreening and the ophthalmic imaging specialists were masked to the results of the ophthalmological examination. Also, the examiners performing the ophthalmological examination were masked to the photoscreening result.

Children were considered to have amblyopia risk factors using the gold-standard ophthalmological examination. Amblyopia risk factor targets are summarized in Table 1, according to previously established guidelines.^10^ Sensitivity, specificity, positive predictive value, and negative predictive value of the smartphone screener in detecting amblyopia risk factors, with its corresponding 95% confidence interval (95%CI), were calculated using the Clopper-Pearson exact method. Analyses were performed both excluding and including the “not gradable” cases as “risk factors identified.”

**Table 1:** Amblyopia risk factors targets in children above 48 months of age.

Amblyopia risk factors				
Hyperopia	Myopia	Astigmatism	Anisometropia	Strabismus
>3.50 D	>1.50 D	>1.50 D	>1.50 D	Manifest misalignment

D: diopters.

## RESULTS

A total of 157 consecutive children were evaluated by photoscreening and were included in the study. Mean age was 6.0±0.5 years (range 5–7). There were slightly more boys than girls (52.9 vs. 47.1%). In 94% of the cases, the software was able to analyze the images and release the result. Compared to the gold-standard ophthalmological examination, sensitivity of the smartphone platform in detecting amblyopia risk factors was 84%, specificity 74%, positive predictive value 86%, and negative predictive value 70%, excluding “not gradable” images. There were nine results classified as “not gradable.” Of these, seven had amblyopia risk factors determined by the comprehensive ophthalmological examination: five had strabismus (four convergent and one divergent), one had high hyperopia, and one had myopia associated with astigmatism and anisometropia. Two children classified as “not gradable” had no amblyopia risk factors. If the “not gradable” images were included as “risk factors identified,” the sensitivity was 85%, specificity 71%, positive predictive value 85%, and negative predictive value did not change (70%). The confidence intervals are provided in Table 2.

**Table 2:** Smartphone photoscreener performance for detecting amblyopia risk factors compared to the comprehensive ophthalmological examination.

Not gradable results	n	Performance metrics			
		Sensitivity, % (95%CI)	Specificity, % (95%CI)	PPV, % (95%CI)	NPV, % (95%CI)
Excluded	148	84 (75–90)	74 (60–85)	86 (77–92)	70 (55–82)
Included as risk factors identified	157	85 (77–91)	71 (57–83)	85 (77–92)	70 (55–82)

PPV: positive predictive value; NPV: negative predictive value; 95%CI: 95% confidence interval.

The prevalence of amblyopia risk factors determined by the comprehensive ophthalmological examination was 67% (105/157). There were 16 false negatives and 13 false positives. Of the false negatives, four had hyperopia (two with associated astigmatism), seven had astigmatism, and five had anisometropia (one hyperopic, one myopic, and three astigmatic). Amblyopia risk factor presentation was unilateral in nine cases and bilateral in seven cases. Of the false positives, eight (62%) children had myopia or astigmatism between 1.25 and 1.50 diopters. The threshold for myopia and astigmatism was ≥1.50. Low hyperopia, myopia, or astigmatism values were found in five additional children.

## DISCUSSION

The key to a successful vision screening tool for pediatricians is to provide high sensitivity and accuracy with ease of use and speed. Photorefraction allows detection of amblyopia risk factors by fast measurements in young children, early enough to achieve favorable treatment outcomes and reduce permanent vision impairment.^17,18^ The GoCheck Kids software enables photorefraction to be done using a smartphone. The sensitivity and specificity of the smartphone platform to detect amblyopia risk factors in our cohort were within the range of traditional instrument-based vision screening technology.^19,20,21,22,23^ This is the first study in a Brazilian population to evaluate the ability of a smartphone photoscreening platform to detect children at risk for amblyopia by comparing smartphone measurements to the gold-standard ophthalmological examination. A recent study compared vision screening using visual acuity and traditional photoscreening and found that both had similar positive predictive values for detecting need for glasses. However, photoscreening took less time, referred more children, and detected a higher number of children with amblyopia than visual acuity testing.^24^ Since the recent release of the GoCheck Kids software version, which includes detection of astigmatism, only one study evaluated the performance of this updated application: Walker et al. in 2020 studied children aged from 6 months to 6 years and found 91% sensitivity, 68% specificity, 57% positive predictive value, and 94% negative predictive value.^25^ The main differences from our results are related to the predictive values. Our lower negative predictive value can be attributed to our higher risk population that had lower number of negative results (52/157; 33%) compared to their study (166/244; 68%). Most of the children from our study were previously screened at school with visual acuity tests performed by local teachers and, therefore, can be considered a high-risk population. Our higher positive predictive value can also be attributed to our higher risk population, but additionally to our participants’ age (mean 72±6 months), which was older than the one in their study (mean 42±22 months). It has been shown that positive predictive value varies significantly according to age, with older children having higher positive predictive value.^16^ One limitation of our study is the lack of comparison with a traditional photoscreener. The smartphone photorefraction results were compared with the comprehensive ophthalmological examination, but not with other screening devices. However, we used the guidelines created by the American Association for Pediatric Ophthalmology and Strabismus for comparing pediatric vision screening modalities.^10^ Comparisons were facilitated by the fact that previous studies evaluating smartphone photoscreening followed the same guidelines. The guiding principles compare the screening results with the child’s comprehensive evaluation in order to determine whether the screening should have prompted a referral to an ophthalmologist or if the child should have passed the screening test.

Vision plays a major role in a child’s global development. Visual maturation in the early years of life is crucial for the formation of permanent neural connections along the ophthalmological pathways. Early detection and treatment of ocular conditions that might impact the maturation of visual functions are important for the proper development of motor skills, reading, social performance, quality of life, and self-esteem.^26,27^ Moreover, in geographic locations with a higher prevalence of untreated eye disorders, there is a significant impairment in the population socioeconomic indicators.^28,29^ Based on those findings, the Brazilian Society of Pediatric Ophthalmology recommends a comprehensive ophthalmological evaluation for all children at an early age. Despite the importance of this recommendation, its implementation is limited by several factors, including lack of access, especially in remote areas; insufficiency of trained pediatric ophthalmologists; and prohibitive health costs. Because instrument-based vision screening technology allows expanding the number of children screened for detection of ocular conditions, photoscreening has been recommended by the American Academy of Pediatrics and the American Association of Pediatric Ophthalmology and Strabismus in children aged 12 months or above.^11,30^ Earlier diagnosis may allow for treatment to be more cost-effective by reducing the number of medical visits required for amblyopia treatment.^10^ Even though one of the main advantages of photoscreening is the ability to screen younger children, who cannot cooperate with visual acuity testing, it also plays an important role for older children, particularly in the setting of high-volume field-based screening, as the one in this study, where quick and efficient measurements are necessary. In photoscreening, both eyes are measured simultaneously in a few seconds only, requiring minimal cooperation from the child. Reliable measurements are achieved in children aged 12 months or above. Since it is not dependent on behavioral responses, it allows screening also in non-verbal older children with developmental delays or learning disabilities who are unable to cooperate with optotype-based visual acuity. Photoscreeners are advantageous, as well, in the setting of busy pediatric services, where visual acuity testing, even in older children, could be challenging and time-consuming. Moreover, photoscreeners are also able to detect abnormalities other than amblyopia, including cataract, pupillary abnormalities, and corneal opacities in children of all ages.^10,11^ The cost of screening devices still can be a limiting factor in some practice settings. Smartphones nowadays are ubiquitous and relatively inexpensive tools. A photoscreener platform using a smartphone with a flash concentrator case can be used by pediatricians to perform vision screening in all annual well-child visits starting with 12 months of age. It is an easy-to-use application with a fast learning curve of approximately 4–5 patients.^25^ To gather additional comparative data, future studies are needed, including different instruments. However, a smartphone photoscreener platform appears to be a promising cost-effective alternative, which could assist pediatricians all over the world and minimize obstacles to vision screening and amblyopia detection.
